# Predicting Kirsten Rat Sarcoma Virus Gene Mutation Status in Patients With Colorectal Cancer by Radiomics Models Based on Multiphasic CT

**DOI:** 10.3389/fonc.2022.848798

**Published:** 2022-06-24

**Authors:** Jianfeng Hu, Xiaoying Xia, Peng Wang, Yu Peng, Jieqiong Liu, Xiaobin Xie, Yuting Liao, Qi Wan, Xinchun Li

**Affiliations:** ^1^Department of Radiology, The First Affiliated Hospital of Guangzhou Medical University, Guangzhou, China; ^2^Department of Pharmaceutical Diagnostics, GE Healthcare, Shanghai, China

**Keywords:** colorectal cancer, computed tomography, radiomics, Kirsten rat sarcoma virus, mutation

## Abstract

**Objective:**

To develop and validate radiomics models based on multiphasic CT in predicting Kirsten rat sarcoma virus (KRAS) gene mutation status in patients with colorectal cancer (CRC).

**Materials and Methods:**

A total of 231 patients with pathologically confirmed CRC were retrospectively enrolled and randomly divided into training(n=184) and test groups(n=47) in a ratio of 4:1. A total of 1316 quantitative radiomics features were extracted from non-contrast phase (NCP), arterial-phase (AP) and venous-phase (VP) CT for each patient. Four steps were applied for feature selection including Spearman correlation analysis, variance threshold, least absolute contraction and selection operator, and multivariate stepwise regression analysis. Clinical and pathological characteristics were also assessed. Subsequently, three classification methods, logistic regression (LR), support vector machine (SVM) and random tree (RT) algorithm, were applied to develop seven groups of prediction models (NCP, AP, VP, AP+VP, AP+VP+NCP, AP&VP, AP&VP&NCP) for KRAS mutation prediction. The performance of these models was evaluated by receiver operating characteristics curve (ROC) analysis.

**Results:**

Among the three groups of single-phase models, the AP model, developed by LR algorithm, showed the best prediction performance with an AUC value of 0.811 (95% CI:0.685–0.938) in the test cohort. Compared with the single-phase models, the dual-phase (AP+VP) model with the LR algorithm showed better prediction performance (AUC=0.826, 95% CI:0.700-0.952). The performance of multiphasic (AP+VP+NCP) model with the LR algorithm (AUC=0.811, 95%CI: 0.679-0.944) is comparable to the model with the SVM algorithm (AUC=0.811, 95%CI: 0.695-0.918) in the test cohort, but the sensitivity, specificity, and accuracy of the multiphasic (AP+VP+NCP) model with the LR algorithm were 0.810, 0.808, 0.809 respectively, which were highest among these seven groups of prediction models in the test cohort.

**Conclusion:**

The CT radiomics models have the potential to predict KRAS mutation in patients with CRC; different phases may affect the predictive efficacy of radiomics model, of which arterial-phase CT is more informative. The combination of multiphasic CT images can further improve the performance of radiomics model.

## Introduction

Colorectal cancer (CRC) is the second most common cancer and the fourth-leading cause of cancer death in China (1). Kirsten Rat Sarcoma virus (KRAS) is the most common mutated oncogene in colorectal cancer, about 30%-45% of patients with CRC have mutations in the KRAS, which is one of the high-risk factors that drive distant metastasis of tumor cells (2). Those patients with CRC who have KRAS mutations have no benefit of the antibody-targeted therapies to the epidermal growth factor receptor (EGFR) (3). Hence, KRAS mutational test has been recommended by the National Comprehensive Cancer Network (NCCN) guidelines for patients with suspected or proven metastatic CRCs for guiding targeted therapy (4).

At present, the gold standard for determinate KRAS mutation status is the pathological examination of tumor tissue in clinical practice (4). However, some patients cannot tolerate biopsy due to its invasiveness, and the insufficient quality of biopsy specimens may hinder efficient and robust mutation testing. In addition, tumor tissues have the characteristics of spatial and temporal heterogeneity (5), which makes biopsy samples may not accurately reflect the tumor genotype expression, especially after multiple treatments (6, 7). Therefore, it would be meaningful for developing a relatively simple and non-invasive method for identifying KRAS mutational status in patients with CRC.

Some non-invasive methods had been used to predict KRAS mutation status in previous studies, and the most used imaging technique was fluorine-18 fludeoxyglucose (18F-FDG) positron emission tomography (PET)-CT (8–11). However, the sample size of these studies was generally small and the research results had been conflicting between different studies (10, 12).

In recent years, radiomics is an emerging technique that has been widely studied in the early diagnosis, efficacy evaluation, and prognosis prediction of tumors (13–16). Previous studies indicated that radiomics has shown great prediction performance and clinical potential for predicting the genetic mutations status of glioma (17, 18), lung cancer (19, 20), and breast cancer (21). In addition, radiomics has been studied in CRC for predicting KRAS mutation (22–27), but most of these studies have only used portal venous phase CT images for radiomics analysis. It is not yet clear whether the non-contrast phase (NCP), arterial phase (AP), venous phase (VP) CT images can be used to predict KRAS mutation in patients with CRC, and the value of the combination of multiphasic radiomics features has yet to be investigated.

Therefore, the aim of our study was to investigate the performance of CT radiomics analysis based on multiphasic CT imaging for predicting KRAS mutation in patients with CRC.

## Material and Methods

### Patients

The study conformed to the provisions of the Declaration of Helsinki (as revised in 2013). Ethical approval is not required for this study as it is based on information collected as part of routine clinical practice. Informed consent was waived because of the retrospective design. We retrospectively analyzed data from patients who were surgically confirmed to have CRC from January 2014 to December 2018. A total of 231 patients met the inclusion criteria for this study. The inclusion criteria and exclusion criteria were shown in **Supplementary Material S1**. All patients were randomly divided into training and test groups in a ratio of 4:1. There were 184 cases in the training group (80 cases of KRAS mutant type, 104 cases of wild type) and 47 cases in the test group (21 cases of KRAS mutant type, 26 cases of wild type).

Baseline clinical characteristics, including age, gender, maximum tumor diameter, levels of carcinoembryonic antigen (CEA), carbohydrate antigen-199 (CA199), and carbohydrate antigen-724(CA724)were collected from the medical records. The pathological characteristics of tumor surgical specimens, including tumor TNM stage, tumor location, and tumor differentiation grade (well, moderately, and poorly differentiated) were assessed as well.

### Identification of KRAS Mutation Status

All surgically resected specimens were processed conventionally by a trained pathologist. DNA was extracted from the formalin-fixed paraffin-embedded (FFPE) tumor tissues by the DNA FFPE Tissue Kit (Xiamen Aide Biological Co., Ltd.). Mutations of KRAS (exons 2, 3, and 4) were analyzed by polymerase chain reaction (PCR) and the amplification refractory mutation system (ARMS) method.

### Image Acquisition and Segmentation

All patients underwent contrast-enhanced abdominal and pelvic CT by using 64-detector or 128-detector row spiral CT systems in our hospital. The CT image acquisition settings are described in **Supplementary Material S2**. All NCP, AP, and VP CT images were retrieved from a picture archiving and communication system (PACS) for image segmentation and analysis except for the portal venous phase CT images.

**Figure 1:**
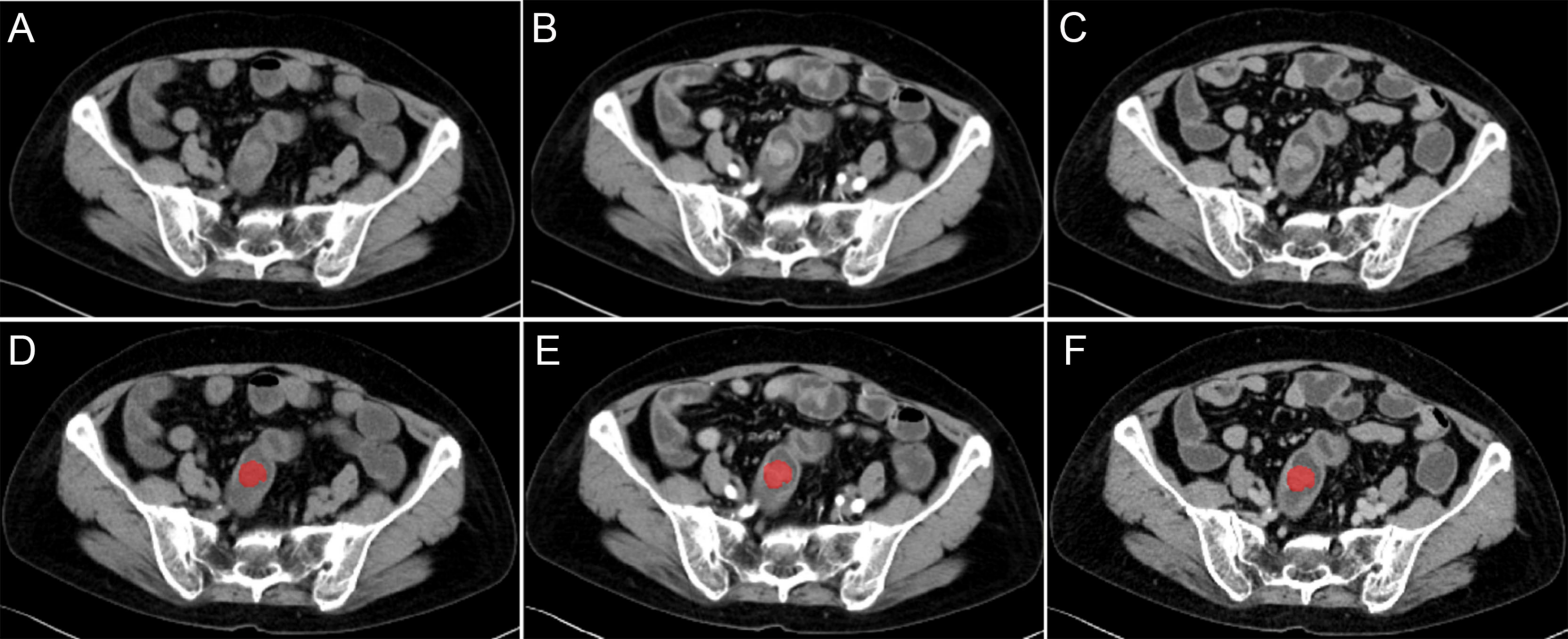
Different CT-phase images used for radiomics analysis. **(A–C)** Images before the preprocessing of non-contrast phase, arterial-phase, and venous-phase. **(D–F)** Images after the preprocessing and delineated along the contour of tumor on the largest slices of tumor.

For the lesion segmentation, the region of interest (ROI) was segmented by using software ITK-SNAP (v.3.8.0; http://www.itksnap.org). Firstly, we manually delineated along the contour of tumors on the largest slices on the VP CT images, excluding the air and feces in the intestinal tract. And the ROIs on the NCP and AP CT images were delineated with reference to that on the VP CT images (**Figure 1**). The ROI of lesions was manually determined by two radiologists with 3 and 8 years of experience, with unanimous agreement. Radiologists were blind to grouping and genetic test results.

### Image Preprocessing and Radiomics Feature Extraction

The original images of all cases and the ROI of lesions were preprocessed by using the AK software (Artificial Intelligence Kit, version 3.3.0, GE healthcare) before radiomics feature extraction. The CT image slice and the ROI was resampled to a uniform pixel dimension size of 1×1×1 mm^3^ by using Linear Interpolation and Nearest Neighbour Interpolation (**Figure 1**).

**Figure 2:**
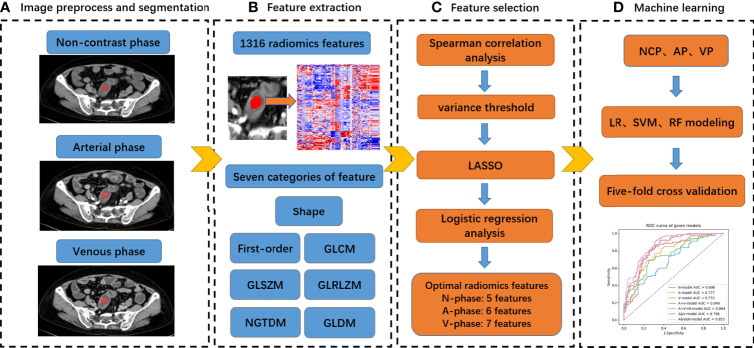
Radiomics analysis workflow of our study.

All radiomics features were extracted using AK software, the detailed information of these features was available in the documentation for PyRadiomics (https://pyradiomics.readthedocs.io/en/latest/features.html), which followed the IBSI radiomics guidelines. Seven categories of feature parameters, including first-order features, shape features, gray level co-occurrence matrix (GLCM), gray level size zone matrix (GLSZM), gray level run length matrix (GLRLM), neighbourhood gray-tone difference matrix (NGTDM), and gray level dependence matrix (GLDM) were selected for feature extraction (**Figure 2**). In addition, Wavelet transform, Laplacian of Gaussian (LoG) and Local binary pattern (LBP) were applied to the original image respectively and yielded a corresponding derived image. Ultimately, a total of 1316 quantitative 2D radiomics features was exacted based on the original image and its corresponding derived image.

### Radiomics Feature Selection and Radiomics Model Building

All cases in the training cohort were used to train the predictive model, while cases in the test cohorts were used to independently evaluate the model’s performance. All radiomics features were imported into the IPMs software (Institute of Precision Medicine Statistics, version 2.4.2, GE healthcare). Before analyses, the missing values were replaced by the median, and the data were standardized by the Z-score method. For the radiomics feature selection, four steps were performed to select the optimal feature subsets for predicting KRAS mutations. First, we used the Spearman correlation analysis. If the correlation coefficient between feature and gene mutation status is small than 0.1, the feature will be eliminated. Second, the Variance threshold method was used to remove features with a variance value lower than 1. Third, the least absolute shrinkage and selection operator (LASSO) algorithm was performed for eliminating the redundancy, this approach can estimate the regression coefficients for every feature and successively shrink them to avoid inflation of the estimated coefficients, resulting in superior predictive performance (28). Fourth, we used multivariate stepwise regression analysis to select the features which were considered to be associated with KRAS mutations. The p-in and p-out of multivariate stepwise analysis were 0.05 and 0.10, respectively. Finally, logistic regression (LR), support vector machine (SVM), and random tree (RT) algorithm were used to build seven groups of prediction models, including the NCP, AP, VP, AP+VP, AP+VP+NCP, AP&VP and AP&VP&NCP models. 5-fold cross-validation was used to select the model with the best performance in the training process. For the SVM and RF, the hyper-parameters of these models were automatic selected by search method, the detailed information of gamma, C, max depth, min samples split, and n estimators were shown in **Table S1**.

### Validation of the Radiomics Model

The receiver operating characteristic (ROC) curve was employed to evaluate the performance of radiomics models for the prediction of KRAS mutation. The area under the curve (including the 95% confidence interval), sensitivity, specificity, and accuracy were also recorded. The calibration curve and the Hosmer-Lemeshow test were used to evaluate the goodness-of-fit of the radiomics model. Decision curve analysis (DCA) was used to evaluate models’ net benefits in different threshold probabilities in the training and test cohort. P<0.05 was considered statistically significant.

### Statistical Analysis

The clinical and pathological characteristics were analyzed by SPSS Statistics 25.0 software, and a two-sided p value of less than 0.05 was statistically considered significant. We used independent samples t-test or Mann-Whitney U test to compare the differences in continuous variables between the patients in different groups, including age and maximum tumor diameter. The differences in categorical variables, including sex, tumor stage, tumor location, tumor differentiation grade, levels of CEA, CA199 and CA724, were assessed using chi-squared or Fisher’s exact tests.

## Result

### Clinical and Pathological Characteristics

There were no significant differences in the clinical and pathological characteristics between the training and the test cohort (p = 0.210-0.879, **Table S2**). The clinical and pathological characteristics in the training and test cohorts are listed in **Table 1**. There were significant differences in TNM stage and M stage between the mutated group and the wild-type group in the training cohort (P < 0.05), but they were not confirmed in the test cohort. There were no significant differences between the mutated group and the wild-type group in both cohorts in terms of age, gender, maximum tumor diameter, tumor location, tumor differentiation grade, T stage, N stage and CEA, CA199, CA724 levels.

**Table 1:** Patient and tumor characteristics in the training and test cohort.

Characteristics	Training cohort			P	Test cohort		P
	Wild-type group (n = 104)		Mutated group (n = 80)		Wild-type group (n = 26)	Mutated group (n = 21)	
Age	61.94 ± 12.27	64.76 ± 12.96		0.133	62.12 ± 13.27	65.52 ± 12.71	0.377
Gender, n (%)							
Male	57 (54.81%)	42 (52.50%)		0.756	16 (61.54%)	10 (47.62%)	0.340
Female	47 (45.19%)	38 (47.50%)			10 (38.46%)	11 (52.38%)	
Tumor location, n (%)							
Ascending colon	30 (28.85%)	28 (35%)		0.260	2 (7.69%)	6 (28.57%)	0.268
Transverse colon	6 (5.77%)	6 (7.5%)			5 (19.23%)	4 (19.05%)	
Descending colon	10 (9.62%)	4 (5%)			2 (7.69%)	2 (9.52%)	
Sigmoid colon	38 (36.54%)	20 (50%)			13 (50%)	5 (23.81%)	
Rectum	20 (19.23%)	22 (27.5%)			4 (15.38%)	4 (19.05%)	
Diameter, cm (Mean ± SD)	5.06 ± 1.85	4.78 ± 1.79		0.296	4.83 ± 1.52	5.44 ± 2.70	0.359
Histologic grade, n (%)							
Poor	12 (11.54%)	11 (13.75%)		0.621	3 (11.54%)	3 (14.29%)	0.645
Moderate	91 (87.50%)	69 (86.25%)			22 (84.62%)	18 (85.71%)	
Well	1 (0.96%)	0 (0.0%)			1 (3.85%)	0 (0.0%)	
TNM stage, n (%)							
I	11 (10.58%)	10 (12.50%)		0.039^*^	3 (11.54%)	4 (19.05%)	0.684
II	51 (49.04%)	24 (30%)			11 (42.31%)	6 (28.57%)	
III	33 (31.73%)	31 (38.75%)			9 (34.62%)	7 (33.33%)	
IV	9 (8.65%)	15 (18.75%)			3 (11.54%)	4 (19.05%)	
T stage, n (%)							
T1	2 (1.92%)	1 (1.25%)		0.909	2 (7.69%)	1 (4.76%)	0.116
T2	14 (13.46%)	13 (16.25%)			2 (7.69%)	3 (14.29%)	
T3	60 (57.69%)	43 (53.75%)			17 (65.38%)	7 (33.33%)	
T4	28 (26.92%)	23 (28.75%)			5 (19.23%)	10 (47.62%)	
N stage, n (%)							
N0	62 (59.62%)	39 (48.75%)		0.317	14 (53.85%)	11 (52.38%)	0.891
N1	26 (25%)	27 (33.75%)			6 (23.08%)	6 (28.57%)	
N2	16 (15.38%)	14 (17.50%)			6 (23.08%)	4 (19.05%)	
M stage, n (%)							
M0	95 (91.35%)	65 (81.25%)		0.044^*^	23 (88.46%)	17 (80.95%)	0.472
M1	9 (8.65%)	15 (18.75%)			3 (11.54%)	4 (19.05%)	
CEA, n (%) ≤ 5 (normal) >5 (abnormal)	57 (54.81%) 47 (45.19%)	35 (43.75%) 45 (56.25%)		0.137	16 (61.54%) 10 (38.46%)	11 (52.38%) 10 (47.62%)	0.528
CA199, n (%) ≤ 39 (normal) >39 (abnormal)	92 (88.46%) 12 (11.54%)	63 (78.75%) 17 (21.25%)		0.073	20 (76.92%) 6 (23.08%)	16 (76.19%) 5 (23.81%)	0.953
CA724, n (%) ≤ 6.9 (normal) >6.9 (abnormal)	91 (87.50%) 13 (12.50%)	69 (86.25%) 11 (13.75%)		0.803	23 (88.46%) 3 (11.54%)	19 (90.48%) 2 (9.52%)	0.824

CEA, carcinoembryonic antigen; CA199, carbohydrate antigen-199; CA724, carbohydrate antigen-724. n, number; SD, standard deviation; *P < 0.05.

### Feature Selection and Radiomics Model Building

**Figure 3:**
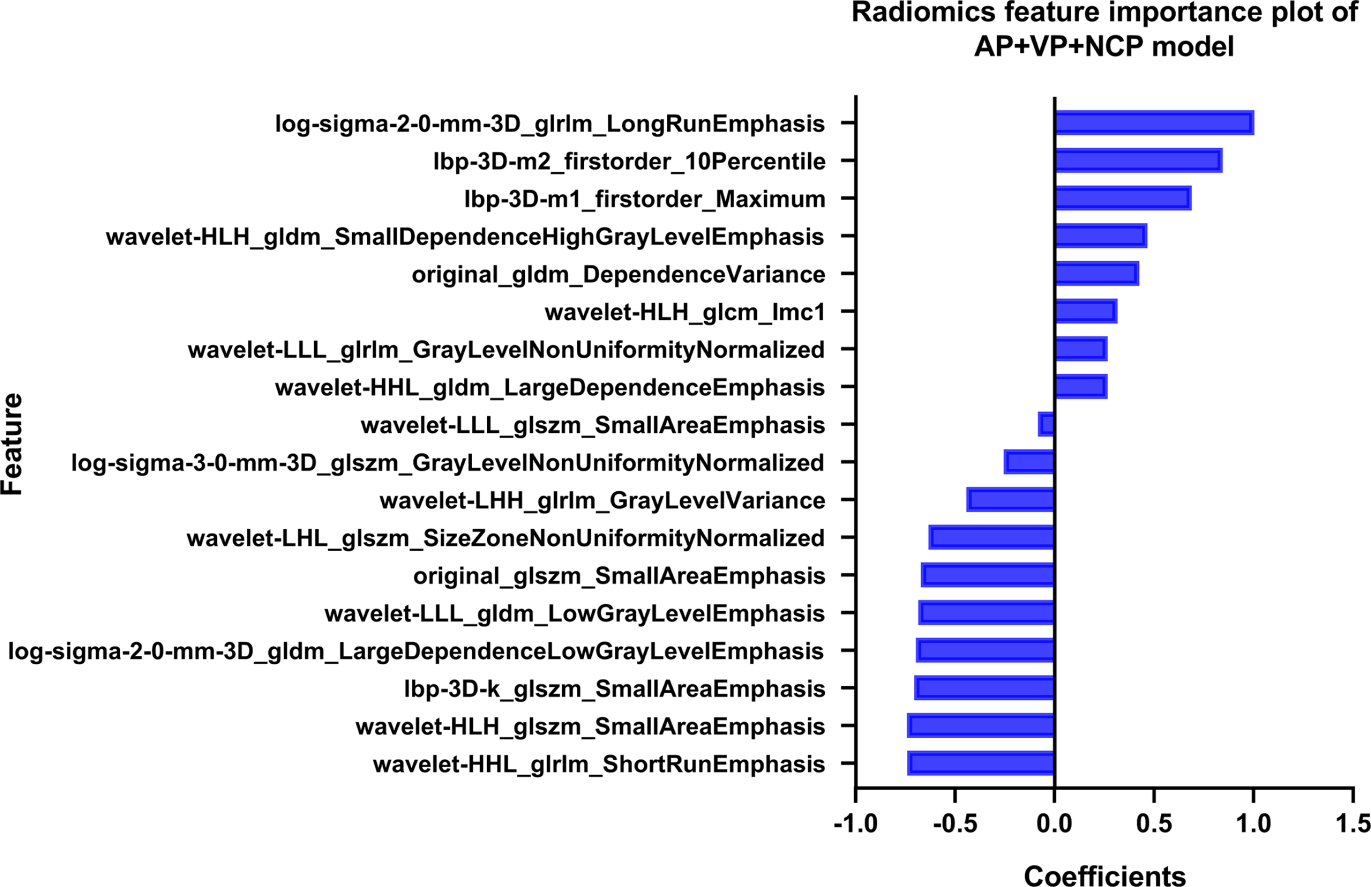
The coefficients of radiomics features in our AP+VP+NCP model developed by LR classifiers.

A total of 1316 radiomics features were extracted from the ROIs of the NCP, AP, and VP CT images for each patient, respectively. After four steps of feature selection, 5, 6 and 7 optimal radiomics features were selected from each phase CT images, respectively (**Table 2**; **Figure 3**). Three groups of single-phase radiomics models were built based on corresponding optimal radiomics features, including the NCP, AP, and VP models. The AP+VP model was built based on 13 (6 + 7) features obtained from the combination of the AP and VP. The AP+VP+NCP model based on 18 (5 + 6+7) features was obtained from the combination of three phases. In addition, we combined the 2632 radiomics features of the AP and VP at first and then four steps of feature selection were implemented, 7 radiomics features were selected to build the AP&VP model. In the same way, 12 radiomics features were selected to build the AP&VP&NCP model.

**Table 2:** Radiomics features for each phase.

CT phase	Category	Names
Non-contrast	GLSZM	[1]original_glszm_SmallAreaEmphasis
	GLDM	[2]wavelet-HHL_gldm_LargeDependenceEmphasis
	GLCM	[3]wavelet-HLH_glcm_Imc1
	GLRLM	[4]wavelet-LLL_glrlm_GrayLevelNonUniformityNormalized
	GLSZM	[5]log-sigma-3-0-mm-3D_glszm_GrayLevelNonUniformityNormalized
arterial	GLRLM	[1]wavelet-HHL_glrlm_ShortRunEmphasis
	GLSZM	[2]lbp-3D-k_glszm_SmallAreaEmphasis
	GLSZM	[3]wavelet-HLH_glszm_SmallAreaEmphasis
	GLDM	[4]original_gldm_DependenceVariance
	GLSZM	[5]wavelet-LLL_glszm_SmallAreaEmphasis
	GLDM	[6]wavelet-HLH_gldm_SmallDependenceHighGrayLevelEmphasis
venous	GLSZM	[1]wavelet-LHL_glszm_SizeZoneNonUniformityNormalize
	First Order	[2]lbp-3D-m1_firstorder_Maximum
	First Order	[3]lbp-3D-m2_firstorder_10Percentile
	GLRLM	[4]log-sigma-2-0-mm-3D_glrlm_LongRunEmphasis
	GLDM	[5]wavelet-LLL_gldm_LowGrayLevelEmphasis
	GLRLM	[6]wavelet-LHH_glrlm_GrayLevelVariance
	GLDM	[7]log-sigma-2-0-mm-3D_gldm_LargeDependenceLowGrayLevelEmphasis

GLCM, gray-level co-occurrence matrix; GLSZM, gray-level size zone matrix; GLRLM, gray level run length matrix; GLDM, gray level dependence matrix.

Finally, seven groups of radiomics models for predicting KRAS mutation were constructed by LR, SVM, and RF classifiers using the above-selected features.

### Predictive Performance of Radiomics Model

**Figure 4:**
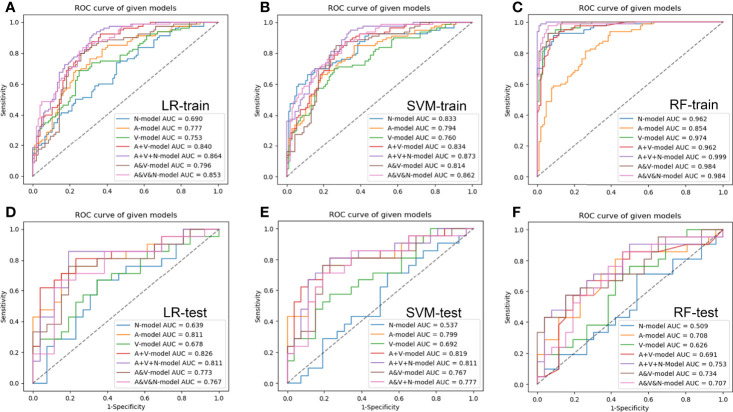
The receiver operating characteristic curves of radiomic models based on different CT-phase images in the training **(A–C)** and test **(D–F)** cohort, respectively. N-model: NCP model; A-model: AP model; V-model: VP model; A+V-model: AP+VP model; A+V+N-model: AP+VP+NCP model; A&V-model: AP&VP model; A&V&N-model: AP&VP&NCP-model.

The results of the seven groups of radiomics model in the training and test cohort were shown in **Figure 4** and **Tables 3**, **4**. Among the three groups of single-phase models, the AP model developed by LR classifiers had the best prediction performance, which had the AUCs of the test cohort was 0.811 (95% CI, 0.685-0.938). Compared to the AP model, the prediction efficiency of the VP model developed by SVM classifiers and NCP model by LR classifiers was relatively lower with an AUC of 0.692(95% CI, 0.556-0.815) and 0.639(95% CI, 0.479-0.800) in the test cohort, respectively. The combined model, including AP+VP model and AP+VP+NCP model, showed an improved performance in comparison with the single-phase model. The AP+VP model developed by LR classifiers showed better prediction performance with an AUC of 0.826(95% CI, 0.700-0.952) in the test cohort. Compared to the AP+VP model, although the AP+VP+NCP model showed no significant improvements in the test cohort with an AUC of 0.811(95% CI, 0.679-0.944) obtained by LR and 0.811(95%CI, 0.695-0.918) by SVM, but the AP+VP+NCP model developed by LR showed better prediction efficiency on the sensitivity, specificity, accuracy, which was 0.810, 0.808 and 0.809, respectively. The AP&VP model developed by LR and AP&VP&NCP model by SVM showed a moderate predictive performance, which had the AUCs of the test cohort was 0.773 (95% CI, 0.650-0.883) and 0.777(95% CI, 0.655-0.889), respectively.

**Table 3:** Performance of the single-phase model in the test cohort.

	parameter	NCP	AP	VP
LR	AUC (95%CI)	0.639 (0.479-0.800)	0.811 (0.685-0.938)	0.678 (0.521-0.834)
	Accuracy	0.617	0.766	0.660
	Sensitivity	0.476	0.762	0.571
	Specificity	0.731	0.769	0.731
SVM	AUC (95%CI)	0.537 (0.393-0.681)	0.799 (0.684-0.900)	0.692 (0.556-0.815)
	Accuracy	0.532	0.766	0.638
	Sensitivity	0.333	0.714	0.381
	Specificity	0.692	0.808	0.846
RF	AUC (95%CI)	0.509 (0.367-0.659)	0.708 (0.574, 0.834)	0.626 (0.494-0.758)
	Accuracy	0.511	0.617	0.532
	Sensitivity	0.333	0.429	0.381
	Specificity	0.654	0.769	0.654

**Table 4:** Performance of the combine phase model in the test cohort.

	parameter	AP+VP	AP+VP+NCP	AP&VP	AP&VP&NCP
LR	AUC (95%CI)	0.826 (0.700-0.952)	0.811 (0.679-0.944)	0.773 (0.650-0.883)	0.767 (0.641, 0.885)
	Accuracy	0.745	0.809	0.723	0.723
	Sensitivity	0.714	0.810	0.762	0.667
	Specificity	0.769	0.808	0.692	0.769
SVM	AUC (95%CI)	0.821 (0.702-0.927)	0.811(0.695-0.918)	0.767 (0.646-0.880)	0.777 (0.655-0.889)
	Accuracy	0.787	0.766	0.766	0.723
	Sensitivity	0.810	0.714	0.714	0.619
	Specificity	0.769	0.808	0.808	0.808
RF	AUC (95%CI)	0.691 (0.557-0.819)	0.753 (0.630-0.867)	0.734 (0.609-0.854)	0.707 (0.582-0.833)
	Accuracy	0.681	0.681	0.702	0.681
	Sensitivity	0.524	0.571	0.571	0.524
	Specificity	0.808	0.769	0.808	0.808

**Figure 5:**
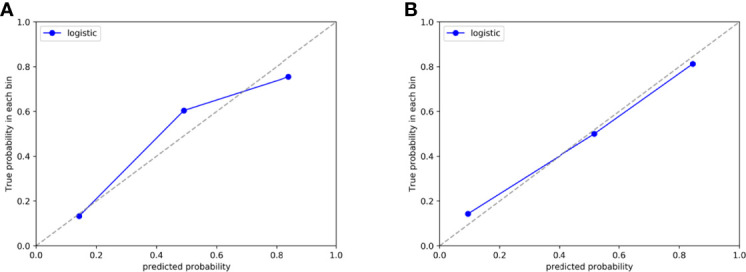
Calibration curve of the AP+VP+NCP model developed by LR classifiers in the training **(A)** and test **(B)** cohort, respectively.

**Figure 6:**
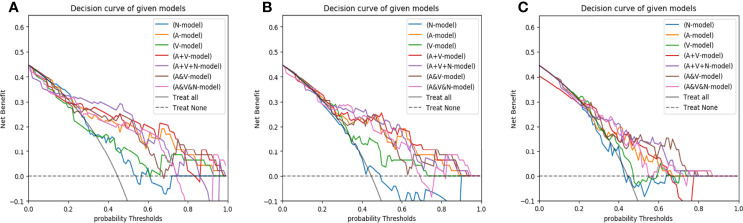
The decision curves of radiomics models developed by three classifiers (**A**, LR; **B**, SVM; **C**, RF) based on different CT-phase images in the test cohort. N-model: NCP model; A-model: AP model; V-model: VP model; A+V-model: AP+VP model; A+V+N-model: AP+VP+NCP model; A&V-model: AP&VP model; A&V&N-model: AP&VP&NCP-model.

The Hosmer-Lemeshow test yielded a non-significant p-value ranging from 0.076 to 0.815 in the LR models. suggesting no departure from the perfect fit. The calibration curve of the multiphasic (AP+VP+NCP) model for KRAS mutation prediction probability shows good accordance between prediction and observation in the training and test cohort (**Figure 5**). The DCA for the seven groups of radiomics models in the test cohort was presented in **Figure 6**. The DCA showed that the AP model, dual-phase model, and multiphasic model showed relatively more area, suggesting the good performance of the radiomics models in terms of clinical application.

## Discussion

In this study, we built seven groups of radiomics models based on different phase CT images for predicting KRAS mutation in patients with CRC. We found that single-phase models have the potential to predict KRAS mutation, with the AP model developed by LR showing the better predictive performance. The model developed by LR showed similar results as SVM except for the NCP model. The predictive performance of the AP+VP and AP+VP+NCP model was further improved compared to that of the single-phase model, and the AP+VP model showed the best predictive performance, but the AP+VP+NCP model showed better predictive performance comprehensively, showing that combing the different phase CT radiomics features could elevate the model’s prediction ability.

There were some previous radiomics studies on the KRAS mutation prediction in CRC. Meng et al. (29) found that radiomic signatures based on multiparametric MRI had the potential to predict KRAS mutation in rectal cancer with an AUC of 0.651 (95% CI, 0.539-0.763). Cui et al. (26)reported that T2WI-based radiomics signature had a moderate performance to predict KRAS mutation in rectal cancer with an AUC of 0.714 (95% CI, 0.602–0.827). The above studies showed an encouraging result for predicting KRAS status by using radiomics, but compare with our study, our best model was the dual-phase (AP+VP) model developed by LR with relatively higher predictive performance, which had an AUCs of 0.826 in the test cohort. Furthermore, as the CT examination is convenient for the patients in clinical practice and also recommended by NCCN guidelines on the management of patients with CRC (4), CT images had been usually used as their research object of radiomics in colorectal cancer. Wu et al. (25)reported that the hand-crafted radiomics signature was associated with the KRAS mutation in CRC with the C-index, sensitivity, and specificity were 0.727, 0.412 and 0.868 in the validation cohort, respectively. Yang et al. (24)found that the proposed CT-based radiomics signature was related to KRAS/NRAS/BRAF mutations with the AUC, sensitivity, and specificity were 0.829, 0.686 and 0.857 in the validation cohort, respectively. Although these studies had higher AUC than that obtained in our study, the sensitivity of these models was relatively low, which may be related to the use of single-phase CT in these studies. While in our study, the single-phase models also showed relatively lower sensitivity, but the multiphasic (AP+VP+NCP) model in our study showed satisfactory predictive performance with the AUC, sensitivity, and specificity were all above 0.8 in the test cohort.

Additionally, many studies in this field have only focused on the portal venous phase CT images (22–25), without investigating the predictive value of radiomics features from other phase CT images for the KRAS mutation in patients with CRC. Some previous studies showed that both unenhanced and contrast-enhanced CT radiomics features have a certain value for reflecting the heterogeneity of tumors (30–32). Badic et al. (30) reported that some radiomics features with moderate correlations between unenhanced and enhanced CT images had complementary prognostic value and were found to be associated with survival in patients with CRC. To the best of our knowledge, this is the first study that predicted KRAS mutation in patients with CRC using different phases CT images. Our results showed that the single-phase model have moderate predictive performance, while the AP+VP and AP+VP+NCP model have further improved predictive performance in comparison with the single-phase model, and the AP+VP+NCP model has more comprehensively predictive performance, suggesting that different phase CT images could provide complementary information for predicting KRAS mutation. We also found that the predictive efficacy of the AP model was better than that of the other single-phase model. It may be that the AP CT image mainly reflects the blood perfusion of the tumor tissue, which may better reflect the tumor microenvironment. Moreover, the predictive performance of the AP+VP and AP+VP+NCP model was slightly higher than those of the AP&VP and AP&VP&NCP model. This may be because the method of feature selection at first and then combined would guarantee that the optimal features of each CT phase can play a role in the combined model.

Choosing a proper classifier can improve the stability and predictive performance of the model. The LR classifier is a linear regression method that had been usually used in many machines learning studies for its good interpretability and suitability to solve dichotomous problem (13, 19, 25). For SVM, it is a robust and effective classifier based on structural risk minimization that had been proved to be a powerful classifier in the previous studies (24, 26). Our previous study had used these two algorithms to build model for distinguishing the solid solitary pulmonary lesion based on T_2_WI images and showed relatively better performance (33). In this study, we could find that the model developed by LR showed similar results as SVM except for the NCP model, and both of these classifiers had moderate predictive performance, it may be that these two classifiers are suitable for solving the problem with a small sample. In addition, although the RF classifier had been showed good performance in other studies (29), which has more hyper-parameters and is a relatively complicated model, the RF classifier showed overfitting in the training and test cohort in our study, it may be that our sample size is relatively small.

For the radiomics features selection, 5, 6, and 7 features were selected from the NCP, AP, and VP, respectively, to form radiomics models, which were mainly derived from GLDM, GLRLM, and GLSZM. The three sets of higher-order radiomics features could quantify the image uniformity and heterogeneity, which were found to be correlated with KRAS mutation in CRC. Among the 18 radiomics features in the AP+VP+NCP model, the log-sigma-glrlm-LongRunEmphasis contributed the most to the detection of KRAS status. The GLRLM refer to quantify gray level runs, which are defined as the length in number of consecutive pixels that have the same gray level value. LongRunEmphasis is a measure of the distribution of long run lengths, with a greater value indicative of longer run lengths and more coarse structural textures within the ROIs, suggesting that the textures of the images with KRAS mutation were more coarse than those without KRAS mutation. Notably, we found that the wavelet features accounted for the largest proportion of the optimal feature set (11/18), indicating that wavelet features have relatively good predictive performance, which is in line with the previous studies (29, 34, 35). Wavelet transform is a common method for multi-scale texture analysis in image processing, which can quantify the heterogeneity within tumors at different scales and extract more texture information (36). In addition, Small AreaEmphasis selected from GLSZM accounted for the largest proportion (3/6) in the radiomics feature set of the AP model, and also appeared in the NCP model (1/5), suggesting that this feature may have good stability in predicting KRAS mutation, which is a measure of the distribution of small size zones, with a greater value indicative of more fine textures within the ROIs.

There were some limitations in this study. First, the present study is a single-center retrospective study, therefore an independent dataset is needed for external validation. Second, the slice thickness of NCP CT images in this study was not completely consistent, however, we have minimized the effect by resampling in the preprocessing process. Finally, 2D segmentation of the tumor was adopted in this study, however, previous studies had shown that the texture analysis results of 2D segmentation and 3D whole-tumor segmentation are similar (37).

In conclusion, our study showed that different phase CT radiomics features could provide different values in predicting KRAS mutations, the combined model, including the dual-phase (AP+VP) model and multiphasic (AP+VP+NCP) model, showed more satisfactory predictive performance compared with the single-phase models, which may suggest that different phase CT images should be considered in radiomics research, rather than single-phase CT image.

## Data Availability Statement

The original contributions presented in the study are included in the article/**Supplementary Material**. Further inquiries can be directed to the corresponding authors.

## Ethics Statement 

The studies involving human participants were reviewed and approved by the First Affiliated Hospital of Guangzhou Medical University. Written informed consent for participation was not required for this study in accordance with the national legislation and the institutional requirements.

## Author Contributions

(I) Conception and design: JH, XXia, and PW; (II) Administrative support: QW and XL; (III) Provision of study materials or patients: YP and JL; (IV) Collection and assembly of data: PW and XXie; (V) Data analysis and interpretation: JH and YL; (VI) Manuscript writing: All authors; (VII) Final approval of manuscript: All authors.

## Funding

This work is supported by the Foundation of Guangzhou Municipal Science and Technology Bureau (202102010253), Guangdong Demonstration Base for Joint Training of Graduate Students (20201), and Open Project Fund of the Sixth Affiliated Hospital of Guangzhou Medical University (2020-11-370).

## Conflict of Interest

Author YL was employed by GE Healthcare, Shanghai, China.

The remaining authors declare that the research was conducted in the absence of any commercial or financial relationships that could be construed as a potential conflict of interest.

## Publisher’s Note

All claims expressed in this article are solely those of the authors and do not necessarily represent those of their affiliated organizations, or those of the publisher, the editors and the reviewers. Any product that may be evaluated in this article, or claim that may be made by its manufacturer, is not guaranteed or endorsed by the publisher.

## References

[1] Sung H Ferlay J Siegel RL Laversanne M Soerjomataram I Jemal A. Global Cancer Statistics 2020: GLOBOCAN Estimates of Incidence and Mortality Worldwide for 36 Cancers in 185 Countries. CA: Cancer J Clin (2021) 71(3):209–49. doi: 10.3322/caac.21660 33538338

[2] Huang D Sun W Zhou Y Li P Chen F Chen H. Mutations of Key Driver Genes in Colorectal Cancer Progression and Metastasis. Cancer metastasis Rev (2018) 37(1):173–87. doi: 10.1007/s10555-017-9726-5 29322354

[3] Van Cutsem E Lenz HJ Köhne CH Heinemann V Tejpar S Melezínek I. Fluorouracil, Leucovorin, and Irinotecan Plus Cetuximab Treatment and RAS Mutations in Colorectal Cancer. J Clin Oncol Off J Am Soc Clin Oncol (2015) 33(7):692–700. doi: 10.1200/jco.2014.59.4812 25605843

[4] Provenzale D Ness RM Llor X Weiss JM Abbadessa B Cooper G. NCCN Guidelines Insights: Colorectal Cancer Screening, Version 2.2020. J Natl Compr Cancer Network JNCCN (2020) 18(10):1312–20. doi: 10.6004/jnccn.2020.0048 PMC8311627 33022639

[5] Burrell RA McGranahan N Bartek J Swanton C. The Causes and Consequences of Genetic Heterogeneity in Cancer Evolution. Nature (2013) 501(7467):338–45. doi: 10.1038/nature12625 24048066

[6] Misale S Yaeger R Hobor S Scala E Janakiraman M Liska D. Emergence of KRAS Mutations and Acquired Resistance to Anti-EGFR Therapy in Colorectal Cancer. Nature (2012) 486(7404):532–6. doi: 10.1038/nature11156 PMC3927413 22722830

[7] Siravegna G Mussolin B Buscarino M Corti G Cassingena A Crisafulli G. Clonal Evolution and Resistance to EGFR Blockade in the Blood of Colorectal Cancer Patients. Nat Med (2015) 21(7):795–801. doi: 10.1038/nm.3870 26030179 PMC4868598

[8] Lv Y Wang X Liang L Wang L Lu J. SUVmax and Metabolic Tumor Volume: Surrogate Image Biomarkers of KRAS Mutation Status in Colorectal Cancer. OncoTargets Ther (2019) 12:2115–21. doi: 10.2147/ott.S196725 PMC6433102 30962693

[9] Chen SW Chiang HC Chen WT Hsieh TC Yen KY Chiang SF. Correlation Between PET/CT Parameters and KRAS Expression in Colorectal Cancer. Clin Nucl Med (2014) 39(8):685–9. doi: 10.1097/rlu.0000000000000481 24978328

[10] Kawada K Toda K Nakamoto Y Iwamoto M Hatano E Chen F. Relationship Between 18f-FDG PET/CT Scans and KRAS Mutations in Metastatic Colorectal Cancer. J Nucl Med Off Publication Soc Nucl Med (2015) 56(9):1322–7. doi: 10.2967/jnumed.115.160614 26135109

[11] Cho A Jo K Hwang SH Lee N Jung M Yun M. Correlation Between KRAS Mutation and (18)F-FDG Uptake in Stage IV Colorectal Cancer. Abdominal Radiol (New York) (2017) 42(6):1621–6. doi: 10.1007/s00261-017-1054-2 28161825

[12] Krikelis D Skoura E Kotoula V Rondogianni P Pianou N Samartzis A. Lack of Association Between KRAS Mutations and 18F-FDG PET/CT in Caucasian Metastatic Colorectal Cancer Patients. Anticancer Res (2014) 34(5):2571–9. 24778079

[13] Huang YQ Liang CH He L Tian J Liang CS Chen X. Development and Validation of a Radiomics Nomogram for Preoperative Prediction of Lymph Node Metastasis in Colorectal Cancer. J Clin Oncol Off J Am Soc Clin Oncol (2016) 34(18):2157–64. doi: 10.1200/jco.2015.65.9128 27138577

[14] Cui Y Yang X Shi Z Yang Z Du X Zhao Z. Radiomics Analysis of Multiparametric MRI for Prediction of Pathological Complete Response to Neoadjuvant Chemoradiotherapy in Locally Advanced Rectal Cancer. Eur Radiol (2019) 29(3):1211–20. doi: 10.1007/s00330-018-5683-9 30128616

[15] Jiang Y Wang H Wu J Chen C Yuan Q Huang W. Noninvasive Imaging Evaluation of Tumor Immune Microenvironment to Predict Outcomes in Gastric Cancer. Ann Oncol Off J Eur Soc Med Oncol (2020) 31(6):760–8. doi: 10.1016/j.annonc.2020.03.295 32240794

[16] Kha QH Le VH Hung TNK Le NQK. Development and Validation of an Efficient MRI Radiomics Signature for Improving the Predictive Performance of 1p/19q Co-Deletion in Lower-Grade Gliomas. Cancers (Basel) (2021) 13(21):5398. doi: 10.3390/cancers13215398 34771562 PMC8582370

[17] Lu CF Hsu FT Hsieh KL Kao YJ Cheng SJ Hsu JB. Machine Learning-Based Radiomics for Molecular Subtyping of Gliomas. Clin Cancer Res an Off J Am Assoc Cancer Res (2018) 24(18):4429–36. doi: 10.1158/1078-0432.Ccr-17-3445 29789422

[18] Le NQK Hung TNK Do DT Lam LHT Dang LH Huynh TT. Radiomics-Based Machine Learning Model for Efficiently Classifying Transcriptome Subtypes in Glioblastoma Patients From MRI. Comput Biol Med (2021) 132:104320. doi: 10.1016/j.compbiomed.2021.104320 33735760

[19] Wang Y Wan Q Xia X Hu J Liao Y Wang P. Value of Radiomics Model Based on Multi-Parametric Magnetic Resonance Imaging in Predicting Epidermal Growth Factor Receptor Mutation Status in Patients With Lung Adenocarcinoma. J Thorac Dis (2021) 13(6):3497–508. doi: 10.21037/jtd-20-3358 PMC8264682 34277045

[20] Le NQK Kha QH Nguyen VH Chen YC Cheng SJ Chen CY. Machine Learning-Based Radiomics Signatures for EGFR and KRAS Mutations Prediction in Non-Small-Cell Lung Cancer. Int J Mol Sci (2021) 22(17):9254. doi: 10.3390/ijms22179254 34502160 PMC8431041

[21] Pinker K Chin J Melsaether AN Morris EA Moy L. Precision Medicine and Radiogenomics in Breast Cancer: New Approaches Toward Diagnosis and Treatment. Radiology (2018) 287(3):732–47. doi: 10.1148/radiol.2018172171 29782246

[22] Li Y Eresen A Shangguan J Yang J Benson AB 3rd Yaghmai V. Preoperative Prediction of Perineural Invasion and KRAS Mutation in Colon Cancer Using Machine Learning. J Cancer Res Clin Oncol (2020) 146(12):3165–74. doi: 10.1007/s00432-020-03354-z PMC11804468 32779023

[23] Taguchi N Oda S Yokota Y Yamamura S Imuta M Tsuchigame T. CT Texture Analysis for the Prediction of KRAS Mutation Status in Colorectal Cancer *via* a Machine Learning Approach. Eur J Radiol (2019) 118:38–43. doi: 10.1016/j.ejrad.2019.06.028 31439256

[24] Yang L Dong D Fang M Zhu Y Zang Y Liu Z. Can CT-Based Radiomics Signature Predict KRAS/NRAS/BRAF Mutations in Colorectal Cancer? Eur Radiol (2018) 28(5):2058–67. doi: 10.1007/s00330-017-5146-8 29335867

[25] Wu X Li Y Chen X Huang Y He L Zhao K. Deep Learning Features Improve the Performance of a Radiomics Signature for Predicting KRAS Status in Patients With Colorectal Cancer. Acad Radiol (2020) 27(11):e254–e62. doi: 10.1016/j.acra.2019.12.007 31982342

[26] Cui Y Liu H Ren J Du X Xin L Li D. Development and Validation of a MRI-Based Radiomics Signature for Prediction of KRAS Mutation in Rectal Cancer. Eur Radiol (2020) 30(4):1948–58. doi: 10.1007/s00330-019-06572-3 31942672

[27] Xu Y Xu Q Ma Y Duan J Zhang H Liu T. Characterizing MRI Features of Rectal Cancers With Different KRAS Status. BMC Cancer (2019) 19(1):1111. doi: 10.1186/s12885-019-6341-6 31727020 PMC6857233

[28] Vasquez MM Hu C Roe DJ Chen Z Halonen M Guerra S. Least Absolute Shrinkage and Selection Operator Type Methods for the Identification of Serum Biomarkers of Overweight and Obesity: Simulation and Application. BMC Med Res Method (2016) 16(1):154. doi: 10.1186/s12874-016-0254-8 PMC5109787 27842498

[29] Meng X Xia W Xie P Zhang R Li W Wang M. Preoperative Radiomic Signature Based on Multiparametric Magnetic Resonance Imaging for Noninvasive Evaluation of Biological Characteristics in Rectal Cancer. Eur Radiol (2019) 29(6):3200–9. doi: 10.1007/s00330-018-5763-x 30413959

[30] Badic B Desseroit MC Hatt M Visvikis D. Potential Complementary Value of Noncontrast and Contrast Enhanced CT Radiomics in Colorectal Cancers. Acad Radiol (2019) 26(4):469–79. doi: 10.1016/j.acra.2018.06.004 30072293

[31] Hodgdon T McInnes MD Schieda N Flood TA Lamb L Thornhill RE. Can Quantitative CT Texture Analysis be Used to Differentiate Fat-Poor Renal Angiomyolipoma From Renal Cell Carcinoma on Unenhanced CT Images? Radiology (2015) 276(3):787–96. doi: 10.1148/radiol.2015142215 25906183

[32] Yang R Wu J Sun L Lai S Xu Y Liu X. Radiomics of Small Renal Masses on Multiphasic CT: Accuracy of Machine Learning-Based Classification Models for the Differentiation of Renal Cell Carcinoma and Angiomyolipoma Without Visible Fat. Eur Radiol (2020) 30(2):1254–63. doi: 10.1007/s00330-019-06384-5 31468159

[33] Wan Q Zhou J Xia X Hu J Wang P Peng Y. Diagnostic Performance of 2D and 3D T2WI-Based Radiomics Features With Machine Learning Algorithms to Distinguish Solid Solitary Pulmonary Lesion. Front Oncol (2021) 11:683587. doi: 10.3389/fonc.2021.683587 34868905 PMC8637439

[34] Kim JY Park JE Jo Y Shim WH Nam SJ Kim JH. Incorporating Diffusion- and Perfusion-Weighted MRI Into a Radiomics Model Improves Diagnostic Performance for Pseudoprogression in Glioblastoma Patients. Neuro-oncology (2019) 21(3):404–14. doi: 10.1093/neuonc/noy133 PMC6380413 30107606

[35] Wu W Parmar C Grossmann P Quackenbush J Lambin P Bussink J. Exploratory Study to Identify Radiomics Classifiers for Lung Cancer Histology. Front Oncol (2016) 6:71. doi: 10.3389/fonc.2016.00071 27064691 PMC4811956

[36] Huang K Aviyente S. Wavelet Feature Selection for Image Classification. IEEE Trans image Process Publ IEEE Signal Process Soc (2008) 17(9):1709–20. doi: 10.1109/tip.2008.2001050 18713675

[37] Lubner MG Stabo N Lubner SJ del Rio AM Song C Halberg RB. CT Textural Analysis of Hepatic Metastatic Colorectal Cancer: Pre-Treatment Tumor Heterogeneity Correlates With Pathology and Clinical Outcomes. Abdominal Imaging (2015) 40(7):2331–7. doi: 10.1007/s00261-015-0438-4 25968046

