# Supplementary material for: Predicting Kirsten Rat Sarcoma Virus Gene Mutation Status in Patients With Colorectal Cancer by Radiomics Models Based on Multiphasic CT
Source: Front Oncol. 2022 Jun 24;12:848798. doi: 10.3389/fonc.2022.848798 (PMC9263192; doi:10.3389/fonc.2022.848798)
Supplement: Supplementary file 1 [file DataSheet_1.docx]

**Supplementary Material**

1. Inclusion and exclusion criteria
2. CT image acquisition
3. Supplementary tables 1
4. Supplementary tables 2
5. **Inclusion and exclusion criteria**

**Inclusion criteria:** (a) patients who pathologically confirmed colorectal cancer; (b) patients who underwent KRAS mutation test; (c) preoperative contrast-enhanced CT including the colorectal cancer; (d) contrast-enhanced CT performed less than 2 weeks before obtaining the pathological tissue sample for the *KRAS* mutation test.

**Exclusion criteria:** (a) patients who underwent radiotherapy, chemotherapy or chemoradiotherapy before obtaining the pathological tissue sample for the KRAS mutation test; (b) preoperative clinical and pathological characteristics incomplete; (c)

the quality of CT images was too poor to be analyzed.

1. **CT image acquisition**

All patients underwent contrast-enhanced abdominal and pelvic CT using 64-detector or 128-detector row spiral CT systems (SOMATOM Perspective, Siemens Healthineers, Germany or SOMATOM Definition AS+, Siemens Healthineers, Germany). The scanning parameters were as follows: 120/130 kv; Automatic tube current; 0.4- or 0.3-second rotation time; matrix, 512×512. After routine unenhanced CT scan, arterial, portal venous and venous-phase contrast-enhanced CT were performed after 20-25s, 60s and 90s delay following intravenous administration of iodinated contrast material (Iohexol Injection, GE Healthcare, USA) at a rate of 4.0 ml/s with a pump injector. The unenhanced CT images was reconstructed with slice thickness of 2/5mm, Contrast-enhanced CT images was reconstructed with reconstruction thickness of 2 mm．

**3. Table S1.** The hyper-parameters of the SVM and RF model

| Model | SVM (RBF) | |  | RF | | |
| --- | --- | --- | --- | --- | --- | --- |
|  | gamma | C |  | max depth | min samples split | n estimators |
| NCP | 0.0400 | 47.1879 |  | 5 | 5 | 20 |
| AP | 0.0069 | 27.3223 |  | 2 | 9 | 25 |
| VP | 0.0265 | 2.9422 |  | 9 | 9 | 15 |
| AP+VP | 0.0015 | 32.6258 |  | 7 | 5 | 5 |
| AP+VP+NCP | 0.0024 | 14.7524 |  | 5 | 3 | 25 |
| AP&VP | 0.0065 | 26.7781 |  | 6 | 9 | 20 |
| AP&VP&NCP | 0.0017 | 50.8173 |  | 5 | 7 | 20 |

RBF, radial basis function.

**4. Table S2.** Demographic differences in the training and test cohorts

| Characteristics | Training cohort  （n=184） | Test cohort（n=47） | P |
| --- | --- | --- | --- |
| Age | 63.17±12.62 | 63.64±12.99 | 0.821 |
| Gender, n (%) |  |  |  |
| Male | 99(53.8%) | 26(55.3%) | 0.852 |
| Female | 85(46.2%) | 21(44.7%) |  |
| Tumor location, n (%) |  |  |  |
| Ascending colon | 54(29.3%) | 12(25.5%) | 0.210 |
| Transverse colon | 13(7.1%) | 8(17.0%) |  |
| Descending colon | 15(8.2%) | 3(6.4%) |  |
| Sigmoid colon | 59(32.1%) | 17(36.2%) |  |
| Rectum | 43(23.4%) | 7(14.9%) |  |
| Diameter, cm (Mean±SD) | 4.94±1.83 | 5.10±2.12 | 0.595 |
| Histologic grade, n (%) |  |  |  |
| Poor | 23(12.5%) | 6(12.8%) | 0.576 |
| Moderate | 160(87.0%) | 40(85.1%) |  |
| Well | 1(0.5%) | 1(2.1%) |  |
| TNM stage, n (%) |  |  |  |
| I | 21(11.4%) | 7(14.9%) | 0.879 |
| II | 75(40.8%) | 17(36.2%) |  |
| III | 64(34.8%) | 16(34.0%) |  |
| IV | 24(13.0%) | 7(14.9%) |  |
| T stage, n (%) |  |  |  |
| T1 | 3(1.6%) | 3(6.4%) | 0.252 |
| T2 | 27(14.7%) | 5(10.6%) |  |
| T3 | 103(56.0%) | 24(51.1%) |  |
| T4 | 51(27.7%) | 15(31.9%) |  |
| N stage, n (%) |  |  |  |
| N0 | 101(54.9%) | 25(53.2%) | 0.706 |
| N1 | 53(28.8%) | 12(25.5%) |  |
| N2 | 30(16.3%) | 10 (21.3%) |  |
| M stage, n (%) |  |  |  |
| M0 | 160(87.0%) | 40(85.1%) | 0.740 |
| M1 | 24(13.0%) | 7(14.9%) |  |
| CEA, n (%)  ≤ 5（normal）  ＞5（abnormal） | 92(50.0%)  92(50.0%) | 27(57.4%)  20(42.6%) | 0.362 |
| CA199, n (%)  ≤ 39（normal）  ＞39（abnormal） | 155(84.2%)  29(15.8%) | 36(76.6%)  11(23.4%) | 0.216 |
| CA724, n (%)  ≤ 6.9（normal）  ＞6.9（abnormal） | 160(87.0%)  24(13.0%) | 42(89.4%)  5 (10.6%) | 0.657 |

CEA, carcinoembryonic antigen; CA199, carbohydrate antigen-199; CA724, carbohydrate antigen-724. Note: n, number; SD, standard deviation.
